# CDKN2A founder mutation in pancreatic ductal adenocarcinoma patients without cutaneous features of Familial Atypical Multiple Mole Melanoma (FAMMM) syndrome

**DOI:** 10.1186/s13053-018-0088-y

**Published:** 2018-03-07

**Authors:** Carol Cremin, Sarah Howard, Lyly Le, Aly Karsan, David F. Schaeffer, Daniel Renouf, Kasmintan A. Schrader

**Affiliations:** 1 Department of Medical Genetics, University of British Columbia, Vancouver, British Columbia Canada; 2 Hereditary Cancer Program, BC Cancer, Vancouver, British Columbia Canada; 3 Department of Medical Oncology, BC Cancer – Surrey, Surrey, British Columbia Canada; 4 Centre for Clinical Genomics, Genome Sciences Centre, BC Cancer Research Centre, Vancouver, British Columbia Canada; 5 Department of Pathology & Laboratory Medicine, University of British Columbia, Vancouver, British Columbia Canada; 6 Pancreas Centre BC, Vancouver, British Columbia Canada; 7 Division of Anatomical Pathology, Vancouver General Hospital, Vancouver, British Columbia Canada; 8 Division of Medical Oncology, BC Cancer, Vancouver, British Columbia Canada; 9 Department of Molecular Oncology, BC Cancer Research Centre, Vancouver, British Columbia Canada

**Keywords:** Pancreatic cancer, *CDKN2A*, Familial atypical multiple mole melanoma syndrome, FAMMM, Melanoma

## Abstract

**Background:**

Approximately 5% to 10% of pancreatic ductal adenocarcinoma (PDAC) has a hereditary basis. In most of these defined hereditary cancer syndromes, PDAC is not the predominant cancer type. Traditional criteria for publicly funded genetic testing typically require the presence of a set combination of the predominant syndrome-associated cancer types in the family history.

We report the identification of a *CDKN2A* pathogenic variant in a PDAC-prone family without the cutaneous features of multiple moles or melanoma that are characteristic of the Familial Atypical Multiple Mole Melanoma (FAMMM) Syndrome identified in a universal testing algorithm for inherited mutations in pancreatic cancer patients.

**Case presentation:**

We present the case of two brothers of English ancestry diagnosed with PDAC within the same 12 month period, at the respective ages of 63 and 64 years of age. Neither brother reported a personal history of multiple moles or melanoma. Family history was positive for two second-degree relatives diagnosed with PDAC but was negative for other cancers or multiple moles in first- and second-degree relatives. Due to the absence of melanoma, this family did not meet provincial criteria for publicly funded genetic testing. Clinical genetic testing offered through a research grant identified a pathogenic variant in the *CDKN2A* gene c.377 T > A (p.Val126Asp). This variant is a North American founder mutation believed to pre-date colonization.

**Conclusions:**

This case reminds clinicians to consider the possibility of a germline *CDKN2A* mutation in families with a high prevalence of PDAC, even in the absence of moles or melanoma. This case supports recent guidelines published by the American College of Medical Genetics and Genomics (ACMG) that genetics referrals are indicated in families with three or more cases of PDAC regardless of other cancer types in the family. A multi-gene panel approach is of particular benefit in diagnosing inherited cancer susceptibility in PDAC-only families.

## Background

Approximately 5% to 10% of pancreatic ductal adenocarcinoma (PDAC) has a hereditary basis with the major genes and syndromes identified thus far outlined in Table 1. In most of these syndromes, PDAC is not the predominant cancer type. Traditional criteria for publicly funded genetic testing typically require the presence of a set combination of the predominant syndrome-associated cancer types in the family history.

**Table 1:** Hereditary pancreatic cancer genes and the 30 gene Color panel. Table listing the major genes associated with hereditary pancreatic adenocarcinoma, the name of the corresponding syndrome and additional cancer risks seen in each syndrome [27]. All of these genes are included in the 30 gene Color Genomics panel. Other genes also included in the 30 gene panel are *MUTYH, MITF, BAP1, PTEN, CDH1, BMPR1A, SMAD4, GREM1, POLD1, POLE, CHEK2, NBN, BARD1, BRIP1, RAD51C, RAD51D* [14]

Gene	Syndrome	Associated Cancers/tumours	Estimated lifetime pancreatic cancer risk (to 70–80 years)	Included on the 30 gene panel
STK11	Peutz Jeghers syndrome	Breast, GI (pancreatic), gynecologic, nasal polyps	11–32%	Yes
PRSS1	Hereditary Pancreatitis	Pancreatic	20%–40%	No
CDKN2A/CDK4	Familial Melanoma (Pancreatic) Syndrome	Melanoma, pancreatic	17%	Yes
BRCA1/BRCA2	Hereditary Breast and Ovarian Cancer syndrome	Breast, ovarian, prostate, male breast, pancreatic	2%–8%	Yes
MLH1, MSH2, MSH6, PMS2, EPCAM	Lynch syndrome	Colon, uterine, ovarian, pancreatic	3%–4%	Yes
APC	Familial Adenomatous Polyposis	Colon, small intestine, desmoid	Elevated, not defined	Yes
TP53	Li Fraumeni syndrome	Breast (young), sarcoma, brain, adrenocortical, leukemia	Elevated, not defined	Yes
PALB2	familial pancreatic cancer	Breast, pancreatic	Elevated, not defined	Yes
ATM	familial pancreatic cancer	Breast, pancreatic	Elevated, not defined	Yes

The genetic heterogeneity of familial pancreatic cancer (FPC), typically defined as the occurrence of pancreatic cancer in two affected first degree relatives, has been well described in the past decade, with mutations in high-penetrance genes such as *BRCA2, CDKN2A, PALB2, STK11* thought to explain only 10%–15% of the familial clustering [1, 2]. In 2015, Zhen et al. reported on a comprehensive analysis of *BRCA1, BRCA2, PALB2,* and *CDKN2A* in a large cohort of FPC kindreds ascertained via the multicenter Pancreatic Cancer Genetic Epidemiology (PACGENE) Consortium and found a mutation prevalence of 8% among 515 FPC patients (41 mutations among the four genes analyzed) [3]. In that study, 2.5% of FPC PDAC patients had a mutation in the *CDKN2A* gene (*n* = 14), which is associated with the Familial Atypical Multiple Mole Melanoma (FAMMM) syndrome. Of note, half of the *CDKN2A* positive FPC kindreds did not have a family history of melanoma. The prevalence of *CDKN2A* mutations increased to 7.8% among PDAC cases with any positive family history of melanoma (*n* = 77).

In keeping with the finding of *CDKN2A* families without a history of melanoma, herein, we report the presentation of a *CDKN2A* pathogenic variant in a PDAC-prone family without the typical cutaneous features of multiple moles or melanoma that are characteristic of the FAMMM syndrome.

### *CDKN2A* gene and FAMMM syndrome

FAMMM syndrome is an autosomal dominant inherited disorder with incomplete penetrance and variable expressivity that results from pathogenic variants in the tumor suppressor gene, *CDKN2A* [4]. It is characterized by multiple nevi (usually in the hundreds), atypical nevi, and melanomas typically diagnosed10–20 years earlier than sporadic melanoma (Table 2). In general, pathogenic variants in the *CDKN2A* gene are associated with a 28% to 67% lifetime risk to age 80 for melanoma and vary by geographic region (compared to a 1.9% risk in the general population) [5, 6]. In some families with FAMMM, the *CDKN2A* mutation is associated with a significantly increased risk for PDAC, though the underlying reasons for this remain to be elucidated [7, 8].

**Table 2:** FAMMM Diagnostic criteria for Familial Atypical Multiple Mole Melanoma syndrome [29]

1. Malignant melanoma in one or more first- or second-degree relatives
2. High total body nevi count (often >50) including some of which are clinically atypical (asymmetric, raised, color variegation present, of variable sizes)
3. Nevi with certain histologic features on microscopy*

*architectural disorder with asymmetry, subepidermal fibroplasia, and lentiginous melanocytic hyperplasia with spindle or epithelioid melanocytes; variable dermal lymphocyte infiltration and the “shouldering" phenomenon.

All three criteria are needed to make a diagnosis

Published recommendations for *CDKN2A* genetic testing include patients with multiple (more than 3) primary melanomas, or families with at least one melanoma and two other instances of melanoma or PDAC in the family with mutation detection rates of 20%–40% in this setting. In the largest familial melanoma data set published, the Melanoma Genetics Consortium (GenoMEL), the presence of pancreatic cancer was strongly predictive for an underlying *CDKN2A* pathogenic variant. Among 466 melanoma-prone families, 185 families (40%) carried mutations in *CDKN2A*. Among the mutation positive families, 49 (28%) reported a family history of PDAC. Of the 66 melanoma families with PDAC, 49 (74%) had a *CDKN2A* mutation. The mutation frequency in melanoma-only families was significantly lower. Only 33% of families without PDAC had a *CDKN2A* mutation [9].

Although PDAC is the second most common cancer occurring in FAMMM syndrome after melanoma, genetic testing of the *CDKN2A* gene is rarely considered in the absence of a family history of moles and melanoma [10]. In British Columbia, the provincial criteria for single-gene *CDKN2A* testing as of January 2017 require the presence of three cases of PDAC/melanoma in close relatives, at least one of which must be melanoma [11]. The recent advent of next-generation sequencing (or massively parallel sequencing), has increased the speed and reduced the cost of genetic testing. The use of multi-gene panels for the assessment of cancer susceptibility is expanding rapidly in clinical practice such that multiple genes can now be assessed at the same time and cost, regardless of whether or not the family history meets traditional criteria for a particular hereditary cancer syndrome [12, 13].

## Case presentation

**Fig. 1:**
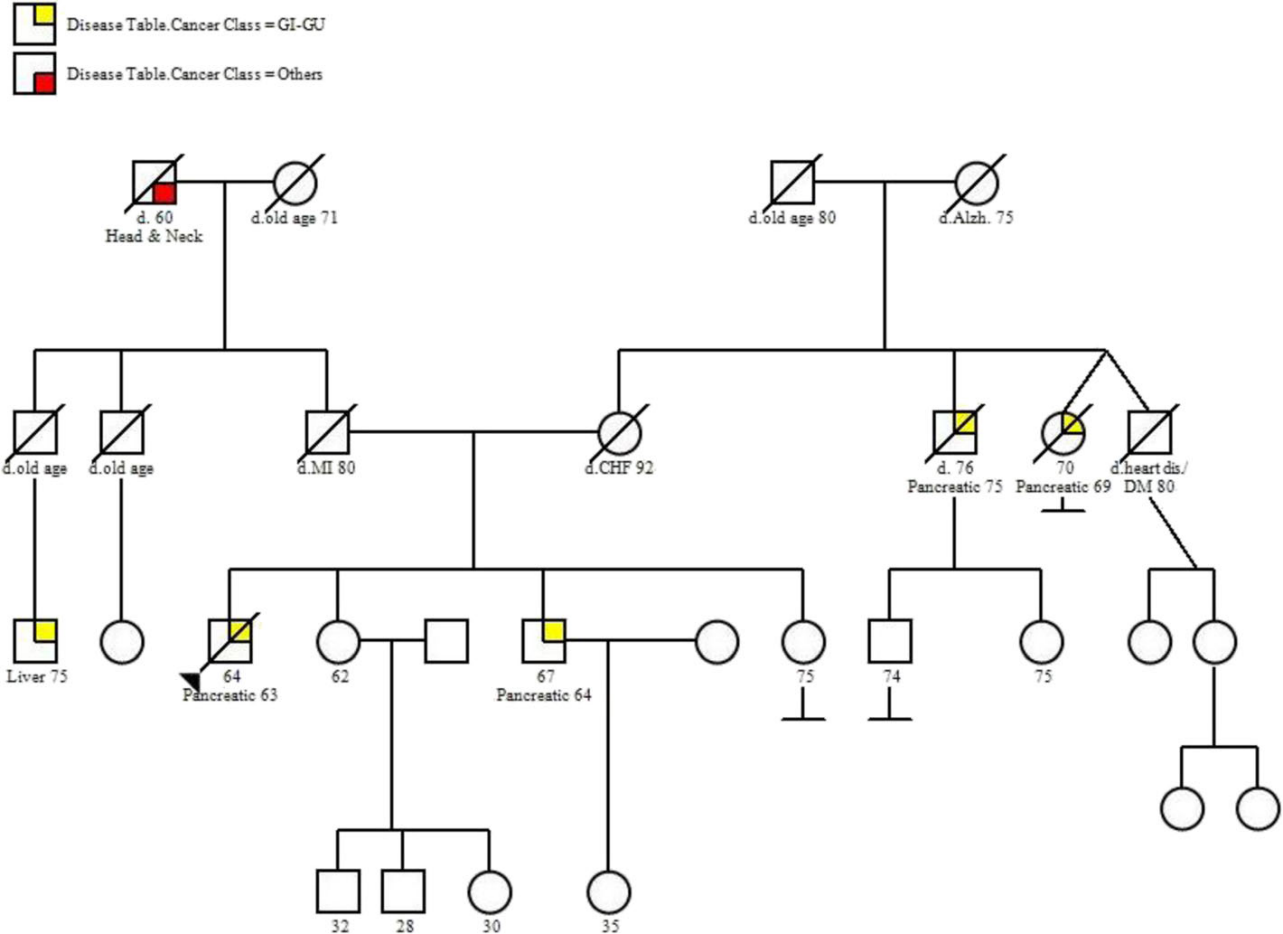
Pedigree. Family history of cancer as reported by the proband (denoted by the arrow), and his brother

A 63 year-old male patient diagnosed with PDAC was referred to the BC Cancer Agency’s Hereditary Cancer Program for hereditary cancer syndrome assessment due to a strong family history of PDAC (Fig. 1). As the starting point for the genetic study of a family, this patient is considered the proband. The family history was noteworthy for the occurrence of PDAC in the proband and his brother, as well as an aunt and uncle on the maternal side. Diagnoses occurred between the ages of 63 and 69. Ancestry was reported as English on both sides.

The proband presented with a three-month history of weight loss and epigastric discomfort. A computerized tomography (CT) scan showed a 2.9 cm lesion in the head of the pancreas. He was deemed to have locally advanced unresectable PDAC and began treatment with a combination of 5-fluorouracil, irinotecan, and oxaliplatin (FOLFIRINOX). His medical history included type 2 diabetes, hypertension, osteoarthritis, gout and hyperlipidemia. He reported being a non-smoker who consumes 6–8 alcoholic drinks per day.

The proband’s brother presented with left upper quadrant pain and underwent an ultrasound as well as a CT scan which revealed the presence of a mass in the pancreatic head and uncinate process. He was deemed to have resectable PDAC and underwent a pancreaticoduodenectomy (Whipple) procedure. Pathology confirmed a 3.5 cm moderately differentiated PDAC. Margins were negative. He was treated with adjuvant gemcitabine for six months. He reported being a non-smoker, rarely consuming alcohol. He denied a significant prior medical history.

During the initial genetic consultation, the proband denied any history of breast, ovarian, colon cancer or melanoma in the family. There was no reported history of pancreatitis, multiple moles, lip freckling or gastrointestinal polyps. In this pancreatic-cancer prone family, none of the diagnoses occurred under the age of 50. The family history of cancer did not meet the current provincial guidelines for hereditary cancer genetic testing [7]. The patient was informed of the options of self-pay genetic testing through one of several genetic testing companies or banking DNA at the BC Cancer Agency should criteria change in the future. He elected to bank a DNA sample and consented to future research.

### Genetic testing

The proband’s brother was referred to the Hereditary Cancer Program six months later at which time a research study had opened that offered index genetic testing to unselected, newly diagnosed cases of PDAC utilizing a commercial-grade genetic test provided by Color Genomics. The test is designed to assess clinically relevant pathogenic variants in 30 genes associated with hereditary cancer risk, including the hereditary breast and ovarian cancer and Lynch syndrome associated genes. Details of their assay are published [14]. After reviewing the advantages and limitations of multi-gene panel testing as well as the implications of possible results, he consented to the research study and provided a saliva sample for genetic testing. Genetic test results revealed a pathogenic missense variant in *CDKN2A*, c.377 T > A (p.Val126Asp). The pathogenic variant is associated with FAMMM syndrome, characterized by an inherited susceptibility primarily to melanoma but also to PDAC. No variants were detected in the remaining genes on the panel.

Due to an inherited predisposition to cancer being diagnosed in his brother, the proband was subsequently seen in follow-up, where he consented to genetic testing. His results were positive for the familial *CDKN2A* pathogenic variant.

## Discussion and conclusions

The frequency of *CDKN2A* pathogenic variants in PDAC families without melanoma has not been well established but was previously considered to be rare. Bartsch et al. (2002) investigated the frequency of *CDKN2A* pathogenic variants in 18 familial pancreatic cancer families and found no pathogenic variants in families without malignant melanoma, compared to two of five families with a history positive for both PDAC and melanoma [15]. McWiliams et al. (2011) describe the challenges in interpreting prevalence and penetrance of germline *CDKN2A* mutations in relation to PDAC given that the mutations have most commonly been described in families where the predominant lesion is cutaneous melanoma [10]. To address this, they studied an unselected PDAC population of 1537 Caucasian patients from the United States and found *CKDN2A* mutations in nine patients (0.6%). Of the 120 cases with a first-degree relative affected with PDAC, four (3.3%) carried mutations, which was significantly higher than in those without a family history. However, five of the nine mutation carriers did not have a first-degree affected relative. The authors estimated the lifetime risk to age 80 of PDAC to be 58% among the 59 first-degree relatives of nine pathogenic variant carriers. This is significantly higher than the 15–25% lifetime PDAC risk reported in melanoma-family studies [16, 17]. However, the authors comment that there was a high proportion of smokers in their study (41%) and that the difference in cancer risk was only evident among ever-smokers. It is interesting to note that the two brothers in this report were non-smokers.

The PACGENE study in 515 FPC kindreds found a nearly five-fold higher prevalence of *CDKN2A* mutations compared to what was seen in the sporadic PDAC population described by McWilliams et al. *(*2.5% compared to 0.6%). Of note, 7 of 14 (50%) of the *CDKN2A* mutation associated FPC kindreds did not have a history of melanoma [3].

The specific pathogenic variant found in this family, p.Val126Asp, is a founder mutation in North American families estimated to have originated approximately 34 to 52 generations ago, pre-dating colonization [18]. The mutation does not appear at high frequency in any other countries besides the United States and Canada. The variant is absent from or extremely rare in population databases [19]. This mutation inhibits the catalytic activity of the cyclin D1/CDK4 and cyclin D1/CDK6 complexes in vitro [20]. Among the seven families reported with this founder mutation in the GenoMEL consortium, PDAC was observed in over 40% and all seven families had a history of melanoma by way of ascertainment [9]. The p.Val126Asp mutation was also described in two PDAC patients in the PACGENE study but details were not provided on the family history of melanoma. In a Dutch study, Harinck et al. described three *CDKN2A* families (two with the Dutch founder mutation c.225_243del, p.Ala76fs and one with a c.19_23dup. pSer8fs mutation) without a history of melanoma and recommended that *CDKN2A* testing be considered even in the absence of reported melanomas [21].

As the use of multi-gene panel testing in patients with PDAC increases, it will be interesting to compare melanoma penetrance in *CDKN2A* families ascertained through FPC criteria compared to *CDKN2A* families ascertained through FAMMM criteria. Further studies are needed to understand what other factors, in addition to the *CDKN2A* pathogenic variant, contribute to the development of PDAC as opposed to melanoma in certain families.

The identification of a germline *CDKN2A* pathogenic variant changes the management of this family in several ways. Publicly funded carrier genetic testing for the known familial *CDKN2A* pathogenic variant is now available to relatives and will clarify cancer risk assessment and management guidelines (Table 3). Of note, the two remaining siblings of this sibship, neither of whom had a personal history of cancer, presented for genetic counseling and tested negative for the founder mutation. From a cancer treatment perspective, an active area of research is the potential predictive role of *CDKN2A* genetic status in terms of estimating sensitivity to CDK4/6 inhibitors [22].

**Table 3:** Familial Atypical Multiple Mole Melanoma (FAMMM) Syndrome Risks and Management. Table listing lifetime cancer risks and management recommendations

Cancer Type	Lifetime Risk	Management [28, 29]
Melanoma	58% - 92% by age 80* [6]	From age 10 or in late adolescence as per family history: - Baseline total body skin examination including scalp, oral mucosa, genital area, and nail, as family members may develop melanoma in their early teens. This screening should also be offered to all first- and some second-degree relatives. Nevi should be examined for ABCDE features of melanoma.** - Total body photography and sequential digital dermoscopy imaging can be useful tools. Examination every 3–6 months initially to ensure nevi stability, then annually. - Thorough total monthly body self-examination should be performed by the patient with assistance from a friend or family member - Routine sun protective behaviors should be reinforced.
Pancreas	17% - 25% [16, 17]	- Avoid smoking - lack of evidence-based data to support pancreatic cancer screening - families should be referred for consideration of clinical research screening programs

*varies with geography

**ABCDE characteristics of the nevus: asymmetric shape, border irregularity, color variegation, diameter greater than 6 mm and elevation or evolution

As with any description of a family history of cancer, one must recognize that the lack of a melanoma in close relatives could be due to incomplete information. Furthermore, family histories are dynamic. However, the consistent reporting from all members of the sibship regarding the lack of any known cases of melanoma in first-, second- or third-degree relatives is compelling and an important presentation to take note of. Indeed, after being encouraged to inform family members about the pathogenic variant and to enquire about any other relatives with melanoma, they became aware of only one single case of melanoma, in a maternal second cousin (fifth-degree relative), in addition to some relatives with moles. To the family’s knowledge, these relatives had not been referred for a formal dermatological assessment.

In recent dermatology literature, a cautionary tone has been expressed concerning the diagnostic pitfall of disregarding family members without nevi as potential carriers of a germline *CDKN2A* mutation. Indeed, this case stresses that the same diagnostic pitfall would also arise by disregarding families without melanoma (+/− or nevi) as potential carriers [23].

There is a strong need to further the evidence-base regarding pancreatic cancer screening in high-risk families. Ideally, this is accomplished through involvement in multidisciplinary clinical research screening programs that are designed to evaluate the ability of both current and new technologies to detect lesions at a stage amenable to therapy. Relatives identified to carry the *CDKN2A* mutation are being invited into a pancreatic cancer screening registry.

Although the two siblings reported here were non-smokers, it is important to stress that the single most important modifier in the risk of PDAC appears to be cigarette smoking. Smoking increases the risk of pancreatic cancer in the general population 1.5 to 5.5-fold [24]. In 2003, Rulyak and others studied pancreatic cancer-prone families and noted that pancreatic cancer developed approximately 10 years earlier in smokers compared to non-smokers [25].

In conclusion, this report describing one of the most common *CDKN2A* mutations reported in North America, in a family without cutaneous features of FAMMM syndrome, should remind clinicians to consider the possibility of germline *CDKN2A* mutations in PDAC-prone families without history of moles or melanoma. This case supports recent guidelines published by American College of Medical Genetics and Genomics (ACMG) that genetics referrals are indicated in families with three or more pancreatic cancers, even in the absence of breast, colon and melanoma cancers [26]. Furthermore, in light of the known genetic heterogeneity, a multi-gene panel approach is of particular benefit in diagnosing inherited cancer susceptibility in PDAC-only families (Table 4).

**Table 4:** ACMG referral indications for cancer predisposition assessment [26]

When to refer for genetic counseling:
A. Pancreatic cancer diagnosed at any age, if any of the following criteria are met.
(i) ≥2 cases of pancreatic cancer in close relatives
(ii) ≥2 cases of breast, ovarian, and/or aggressive prostate cancer in close relatives
(iii) Ashkenazi Jewish ancestry
B. Pancreatic cancer and ≥1 Peutz Jeghers type polyp in the same person
C. Pancreatic cancer and two additional cases of any Lynch Syndrome associated cancer in the same person or in close relatives
D. ≥3 cases of pancreatic cancer and/or melanoma in close relatives
E. Pancreatic cancer and melanoma in the same person
F. Unaffected but with a family history of:
(i) Ashkenazi Jewish ancestry and pancreatic cancer at any age in a close relative
(ii) three or more cases of breast, ovarian, pancreatic and/or aggressive prostate cancer in close relatives
(iii) three or more cases of pancreatic cancer and/or melanoma.

## Abbreviations

ACMG: American College of Medical Genetics and Genomics
CT: computerized tomography
FAMMM: Familial Atypical Multiple Mole Melanoma
FOLFIRINOX: leucovorin, 5- fluorouracil, irinotecan and oxaliplatin
GenoMEL: Melanoma Genetics Consortium
PDAC: pancreatic ductal adenocarcinoma
